# Addressing Emerging Risks: Scientific and Regulatory Challenges Associated with Environmentally Persistent Free Radicals

**DOI:** 10.3390/ijerph13060573

**Published:** 2016-06-08

**Authors:** Tammy R. Dugas, Slawomir Lomnicki, Stephania A. Cormier, Barry Dellinger, Margaret Reams

**Affiliations:** 1 Department of Comparative Biomedical Sciences, LSU School of Veterinary Medicine, Baton Rouge, LA 70803, USA; tammydugas@lsu.edu; 2 Department of Environmental Sciences, Louisiana State University and A & M College, Baton Rouge, LA 70803, USA; slomni1@lsu.edu; 3 Department of Pediatrics, University of Tennessee Health Sciences Center and Children’s Foundation Research Institute, Le Bonheur Children’s Hospital, Memphis, TN 38103, USA; scormier@uthsc.edu; 4 Department of Chemistry, Louisiana State University and A & M College, Baton Rouge, LA 70803, USA; barryd@lsu.edu

**Keywords:** particulate matter, free radicals, PM_2.5_, combustion, incineration, superfund sites

## Abstract

Airborne fine and ultrafine particulate matter (PM) are often generated through widely-used thermal processes such as the combustion of fuels or the thermal decomposition of waste. Residents near Superfund sites are exposed to PM through the inhalation of windblown dust, ingestion of soil and sediments, and inhalation of emissions from the on-site thermal treatment of contaminated soils. Epidemiological evidence supports a link between exposure to airborne PM and an increased risk of cardiovascular and pulmonary diseases. It is well-known that during combustion processes, incomplete combustion can lead to the production of organic pollutants that can adsorb to the surface of PM. Recent studies have demonstrated that their interaction with metal centers can lead to the generation of a surface stabilized metal-radical complex capable of redox cycling to produce ROS. Moreover, these free radicals can persist in the environment, hence their designation as Environmentally Persistent Free Radicals (EPFR). EPFR has been demonstrated in both ambient air PM_2.5_ (diameter < 2.5 µm) and in PM from a variety of combustion sources. Thus, low-temperature, thermal treatment of soils can potentially increase the concentration of EPFR in areas in and around Superfund sites. In this review, we will outline the evidence to date supporting EPFR formation and its environmental significance. Furthermore, we will address the lack of methodologies for specifically addressing its risk assessment and challenges associated with regulating this new, emerging contaminant.

## 1. Introduction

Residents near Superfund sites are exposed to fine and ultrafine particles through a variety of routes, including inhalation of windblown dust, ingestion of soil and sediments, and inhalation of emissions from the on-site thermal treatment of contaminants. Since the particles may originate from sites contaminated with hazardous substances, they may also be contaminated. While windblown dust and soils contain a large fraction of coarse particles, designated by the US-EPA as PM_10_ (particles with a diameter of less than 10 microns), they also contain fine particles, or PM_2.5_ (with a diameter of <2.5 microns), and ultrafine or nanoparticles, PM_0.1_ (diameter of <0.1 microns or <100 nm). With publication of epidemiological data since 1990 linking exposure to PM_2.5_ with cardiopulmonary diseases, the impact of PM_2.5_ and PM_0.1_ on human health has become a major environmental issue [1,2,3].

In general, the emission of organic pollutants during thermal treatment processes results from poor mixing of gasses and formation of oxygen-starved pockets in various areas of the flame, including the post flame and cool zones of the thermal treatment process. Combustion and/or oxidation are incomplete in these areas, resulting in the formation of so-called “products of incomplete combustion”. Semi-volatile and nonvolatile organic pollutants have long been thought to associate with particulate matter through adsorption to the surface or within the particles’ pore system and cracks. These organic compounds can also rapidly react with the surfaces of particles at moderately elevated temperatures in thermal and combustion systems. It has been shown that such reactions are a major route to the formation of polychlorinated dibenzo-*p*-dioxins and polychlorinated dibenzofurans, popularly known as “Dioxins” [4,5,6]. During such reactions, the adsorbing molecules interact with metal centers, leading to an electron transfer process and formation of surface stabilized metal-radical complexes [7,8]. Upon emission with particulate matter, such entities are resistant to further oxidation in the ambient atmosphere, can persist in the environment for days, weeks or longer [9,10] and are thus referred to as Environmentally Persistent Free Radicals (EPFRs). These EPFRs can be generated through combustion of a variety of source materials and typically elicit a single broad, unstructured electron paramagnetic resonance signal characteristic of an oxygen-centered radical, e.g., *g* value ≈ 2.003 (see Table 1) [11,12,13,14]. Indeed, recent studies have indicated the ubiquitous presence of similar radical signatures in ambient air PM_2.5_ and have attributed its presence with EPFRs, similar to those formed in thermal processes [15].

Early research demonstrated EPFR formation for thermal processes and combustion-derived PM [11,12,13,14]. Though there are no data as yet for the levels of EPFRs in PM generated during the thermal remediation of a Superfund site, soils collected in and around them have revealed the presence of EPFRs, and these levels were 2–30-fold higher than in soils collected in areas removed from the sites [18]. This is not unexpected, since soils at Superfund sites should contain many of the same constituents of PM derived from its thermal remediation, including transition metals and organic pollutants. In fact, at sites shown contaminated with pentachlorophenol [18,19] and PAHs [18], analysis of collected soils using electron paramagnetic resonance revealed EPFRs of similar characteristics as that generated by the combustion of similar hydrocarbons [20]. While combustion processes involved in thermal remediation produce vast quantities of EPFRs with reaction times of seconds, in soils, similar EPFRs are likely formed, albeit at reaction times on the order of years rather than seconds. The ability to form EPFRs in ambient conditions was further supported by their detection in tar balls collected at the Gulf of Mexico shores of Louisiana and Florida after the Deepwater Horizon platform oil spill [21]. On the other hand, laboratory studies have shown that low temperature thermal treatment of contaminated soils can increase the concentration of EPFRs [22]. In this review, we will summarize the evidence for the presence of EPFRs in a wide range of environmental matrices, e.g., soils, particulate matter, *etc.* Further, we will outline the evidence that despite a lack of risk assessment strategies adapted for these pollutant-particle systems, EPFRs are an emerging contaminant that deserves attention, research focus, and efforts in the development of appropriate methodologies for real-time monitoring, risk assessment and public policy.

## 2. Epidemiological Studies of Hazardous Waste Incineration and Other Thermal Processes

Epidemiological data now strongly suggest an association between elevations in particulate matter emissions and a number of cardiovascular and respiratory events. Specifically, air pollution including PM has been shown to both exacerbate [23] and increase the onset of asthma [24,25,26]. In the Framingham Heart Study, short-term exposures to PM_2.5_, even within EPA standards (PM_2.5_ < 12 μg/m^3^ annually), were shown associated with decreased lung function [27]. Moreover, epidemiological data suggest a link between PM exposures and cardiovascular events and mortality [28]. For example, results of the ESCAPE (European Study of Cohorts for Air Pollution Effects) study revealed a 13% increase in coronary events for each 5 µg/m^3^ increase in annual mean PM_2.5_ exposures [29]. In a population-based cohort study in Canada, incident hypertension was shown to be associated with elevations in PM_2.5_, with a 13% increase in risk for every 10 μg/m^3^ increase in PM_2.5_ levels [30]. Using Medicare program hospital admissions coupled to spatial resolution of PM exposure data within 20 km of EPA air monitoring stations, Kloog *et al.* [2], showed that admissions for respiratory and cardiovascular events, such as those related to valvular disease, stroke, ischemic heart disease and chronic obstructive pulmonary disease, were associated with elevations in PM_2.5_ exposures.

Given reports that ~40%–70% of airborne fine particles are derived from emissions from combustion sources [31,32], it is reasonable to assume that combustion-derived PM plays a role in epidemiological data linking PM exposures and cardiopulmonary diseases. In fact, some studies have directly determined the contribution of combustion-derived PM in these health effects outcomes. A notable example is the association between decreased lung function and increased prevalence of chronic obstructive pulmonary disease (COPD) and respiratory infections reported in populations exposed to particulates from the combustion of biomass fuels for cooking [33,34]. In another example, the National Particle Component Toxicity (NPACT) initiative sought not only to determine an association between PM levels and health effects, but also utilized data from the EPA’s Chemical Speciation Network (CSN) to compare these health outcomes across putative sources of particulates [3]. Findings from this study suggested that PM_2.5_ derived from fossil fuel combustion was associated with both short-term and long-term health effects. Moreover, PM_2.5_ originating from residual oil combustion and traffic sources were associated with short-term health effects, while PM_2.5_ derived from coal combustion was correlated with long-term health consequences [3].

As has been reviewed extensively by others, [35,36] the initiation of cardiovascular diseases such as atherosclerosis typically results from cycles of oxidative stress and inflammation within affected cells. Given the known relationship between certain heavy metals and their ability to initiate oxidative stress [37], many laboratories have sought to elucidate whether the metal content of PM has a role in exacerbating PM-related cardiovascular diseases. As an example, studies have shown elevations in ischemic heart disease in trucking industry workers exposed chronically to particulate emissions [38], and PM emitted from diesel engines is associated with a wide range of heavy metals [39,40]. Moreover, these exposures were associated with elevated plasma biomarkers for oxidative stress and inflammation [41], suggesting that these factors may play a role in the biological mechanisms of action of these PM. In addition, plasma obtained from workers exposed to metal-rich PM, particularly iron, exhibited elevated levels of oxidative stress markers [42]. The investigators hypothesize that the oxidative stress condition induced by inhalation of metal-containing PM serves as a critical systemic link between PM exposures and its associated increased risk of cardiovascular disease.

Other epidemiological and basic science research studies have revealed disparate findings with respect to the role of metals in mediating the health effects of PM. For example, comparing biological specimens obtained from volunteers in cities in Japan and China with comparable ambient air PM_2.5_ levels but dramatically different metal content, Niu *et al.*, showed that exposures to PM_2.5_ containing Ni, Cu, As, and Se produced more dramatic reductions in circulating endothelial progenitor cells but increased levels of inflammatory cytokines and other markers [43]. On the other hand, analysis from two large cohorts revealed no relationship between cardiovascular mortality and the levels of Cu, Fe, K, N, S, Si, Va, Zn present in PM [44]. Conversely, in a study assessing incidences of low birth weights in California, increased rates were associated with PM_2.5_ containing specific metals, such as Va, Fe, Ti, Mn, and Cu [45]. Thus, our understanding of the role of the metal component of PM in its health risk of exposure is incomplete. As will be explained below, elucidating its contribution to the health risk of PM exposure may require delving beyond its behavior as an individual unit to that of its role in a more complex pollutant-particle system. Specifically, we believe that the metal is important as an entity necessary to form the pollutant-particle systems responsible for EPFR formation, and that different metals impart differences in radical formation and stability in the environment and in the host.

## 3. The Case for Environmentally Persistent Free Radicals

**Figure 1:**
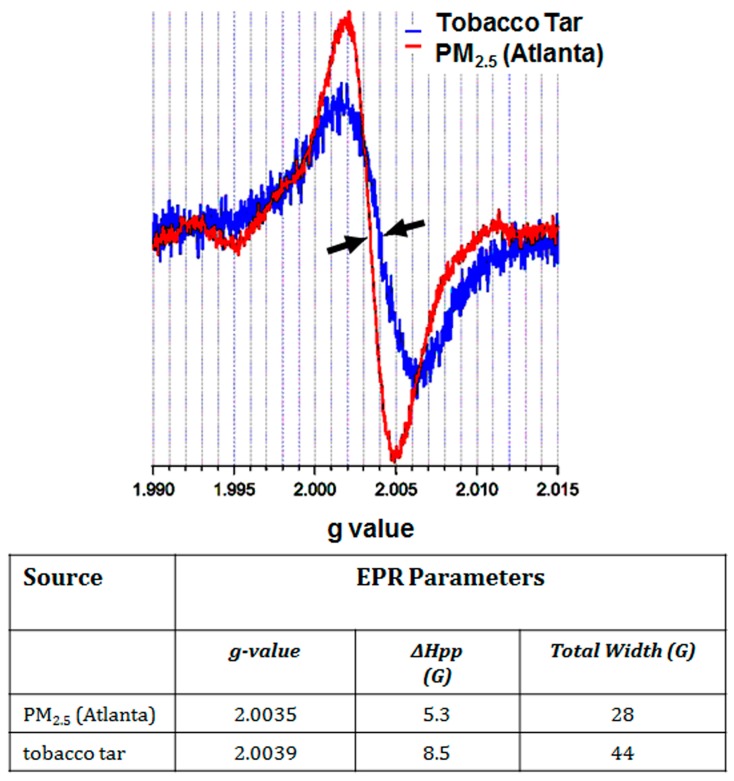
EPR spectra of ambient air PM_2.5_ (Atlanta) and cigarette tar, with a centerfield of 3550 Gauss. In both cases, the observed spectra is typical of an EPFR. The larger width of the tobacco tar signal is indicative of more complex convolution due to the presence of multiple radicals. Arrows point to the g-value for each spectrum.

Combustion of organic materials is generally known to generate free radicals due to gas phase reactions (at 600 °C–1200 °C). These radicals evolve from the thermal dissociation/scission of chemical bonds and the reaction of these radicals with other radicals and molecules present in the plasma region of the flame, *i.e.*, that which contains ionized gases. These radicals are very reactive and short-lived, with a half-life of nano- to- milliseconds, depending on the particular species. Thus, the discovery of EPFRs and elucidation of mechanisms for their formation [8] has created a new paradigm for the perception of combustion-borne radicals as long-lived entities. Studies utilizing laboratory simulations of a combustion reactor showed that in the cool-zone region, where temperatures are 100 °C–600 °C, chemicals on the surfaces of particles condense to form additional pollutants and stable free radicals detected using Electron Paramagnetic Resonance (EPR) [46]. Recognition of the presence of long-lived carbon-centered radicals in solid matrices such coals, chars, and soots dates back to the 1950’s [47,48] and were associated with delocalized electrons in a polyaromatic organic polymer, although with differing spectral characteristics (*g* ~2.002–2.003). Long-lived radicals in cigarette tar exhibiting spectral characteristics similar to EPFRs were identified as semiquinone radicals [49,50]. These semiquinone radicals were shown associated with a quinone/hydroquinone redox cycle capable of producing reactive oxygen species (ROS) [49,50] and an ability to induce DNA damage [49,51]. EPR studies conducted as early as 1982 demonstrated similar free radical species in diesel exhaust particles [52]. Since then, numerous laboratories have demonstrated the presence of long-lived free radicals formed in combustion products from a wide range of sources, ranging from wood, coal and biochar, to diesel and gasoline exhaust products, to ambient PM and polymer waste products, as is highlighted in Table 1 [11,12,13,14,17,53]. Importantly, PM_2.5_ examined from 5 different sites across the country (LA, AZ, CA, NC, PA) demonstrated levels of free radicals comparable to that of cigarette smoke—10^16^–10^17^ radicals/gram [54]. As is illustrated in Figure 1, which depicts an EPR spectrum of ambient PM from Atlanta compared to that of cigarette tar, the spectral parameters of these persistent free radicals, including *g*-values in the range of 2.0031–2.004, were similar to phenoxyl/semiquinone radicals, and this radical could be detected in samples stored for several months.

## 4. Mechanisms for EPFR Formation

The mechanism for EPFR formation involves the chemisorption of undestroyed parent molecules, incomplete combustion byproducts or molecules formed *de novo* onto freshly incepted particles, along with metal center domains. Studies aimed at characterizing EPFR formation have shown that incomplete combustion of carbon, carbon and chlorine, carbon and nitrogen and carbon and bromine forms a number of products, including small hydrocarbons such as ethylene, acetylene and others, but also benzene, phenols, and PAHs, including their chlorinated and brominated derivatives [55,56,57]. These compounds can adsorb onto condensed refractory metal oxides at temperatures ranging from 120 to 900 °C, forming particulate matter and culminating in the formation of pollutant particle systems that include EPFRs capable of producing ROS. In support of this claim, virtually every metal and organic compound observed at increased body burden downwind of incinerators has been found to be contained within particulate emissions from these incinerators, as are a number of known carcinogens and other toxic compounds [58,59,60].

**Figure 2:**
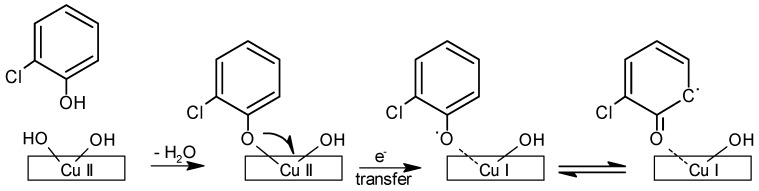
Interaction of a pollutant with a metal oxide cluster. In this representation, monochlorphenol is chemisorbed to the surface of the particle by the elimination of a molecule of water. A 1-electron transfer then results in Cu II reduction to Cu I and the formation of a surface-stabilized, oxygen-centered radical. It is resonance with a carbon-centered radical(s) on the ring further stabilizes the radical.

Most aromatic compounds will chemisorb to the surface of metal-oxide-containing particulate matter under post-combustion, cool-zone conditions (120 °C–400 °C). Chemisorption is defined as the formation of a chemical bond between the particle and a pollutant, resulting in a new metal-pollutant entity that will exist until a subsequent chemical reaction occurs to separate or destroy them. Many transition metal oxides can be easily reduced by a chemisorbed organic (Figure 2) [13]. Using X-ray spectroscopy and FTIR methodologies, as well as surrogate systems for copper-containing fly ash, it was demonstrated that in the post-combustion zone, organic compounds react with surface-dispersed CuO to generate phenolate products, while at the same time, Cu(II) is reduced to Cu(I) [61]. In the process of reducing the metal, a surface-associated organic free radical is formed. This research has revealed that the association of the free radical with the surface of the metal-containing particle stabilizes the radical [7]. In fact, these radicals are stable for days in air at room temperature. The precise mechanism promoting their stabilization is not clear. We postulate that their resistance to oxidation in air is mainly due to the electronic resonance of the radical and the distribution of the unpaired electron over the entire molecule and extending further to the metal center.

We have now completed multiple studies indicating these EPFRs form and persist in soils from contaminated Superfund sites, PM generated from the thermal treatment of hazardous substances including e-wastes (unpublished data), airborne fine PM, and even e-cigarette aerosols [62]. We have determined the conditions under which persistent free radicals are generated and can reproduce these conditions in the laboratory to produce surrogate systems for biomedical, chemical and physical studies.

Superfund sites, in particular, are a rich source of organics and metals that together can form matrices containing EPFRs. As an example, it has already been shown that semiquinone radicals form from the oxidation of PCBs [63], and PCBs are a common contaminant at Superfund sites. Moreover, sediments and soils contaminated with pentachlorophenol in and around a Superfund wood treatment site generated EPFRs [64]. Follow-up studies also indicated the presence of EPFRs in soil and sediment samples from Superfund sites in Montana and Washington [18]. Thus, EPFRs can be presumed a common species in Superfund sites nationwide.

## 5. EPFR-Induced Production of Reaction Oxygen Species (ROS) and Their Potential Health Effects

As mentioned earlier, metal and organic compounds observed at increased body burden downwind of incinerators have been found to be contained within particulate emissions generated by these nearby incinerators [58,59,60]. Some have argued that although these metals are accessible for human exposures through even non-inhalation pathways—*i.e.*, via water, foliage, soils, *etc.*—levels observed in those sources are likely more reflective of ordinary ambient air exposures [65]. Nevertheless, given that combustion-derived metals may exist as a component of an EPFR, these metal-pollutant complexes may exhibit toxicities that are much greater than, or “more-than-additive”, compared to that of their metal and organic components, per se. Thus, risks associated with exposure to these combustion-derived metals may be misinterpreted.

Studies published in the last decade have suggested the EPFR’s ability to generate ROS such as hydroxyl radical, and consequently, their ability to induce the oxidation of biomolecules *in vitro* (Table 2) [17,66,67,68]. Early studies examined the ability of airborne PM to induce oxidative DNA damage [13,17,53,67]. Valavanidis *et al.*, showed than upon suspension together with H_2_O_2_, particulates such as PM_10_, PM_2.5_, diesel and gasoline exhaust particles and wood smoke soot induced the hydroxylation of 2′-deoxyguanosine in a manner correlated with its content of both transition metals and a free radical species detected by EPR [53]. Later that same year, though, the group reported that these same PM were capable of producing ROS independent of exogenously added hydrogen peroxide [13]. EPR characterization of the PM demonstrated a single, broad signal at *g* = 2.0036, indicative of a semiquinone radical. The authors argue that the broadness of the signal suggested a group of semiquinone radicals in a polymeric matrix. Alaghmand and Blough likewise found that suspensions of airborne PM from a variety of sources, but particularly that derived from diesel exhaust (DEP), generated hydroxyl radical in a metal ion-dependent manner [69]. Biological electron donors such as NADPH further stimulated hydroxyl radical production, and through experiments involving centrifugation and filtration, they showed that hydroxyl radical formation was likely due to particle surface reactions rather than reactions occurring in solution via solubilized PM components [69]. DiStephano *et al.*, also found that DEP and PM collected at sites across California were capable of generating hydroxyl radical in a manner correlated with its Cu content [70]. Similar to studies by Alaghmand and Blough, hydroxyl radical production was further stimulated by co-incubation with an electron donor, in this case, ascorbate [70]. In sum, these studies suggest that PM is a complex mixture, but its ability to redox cycle to produce ROS likely derives from an interaction between its semiquinone radical and transition metal content, presumably through Fenton-type reactions [71] on the particle surface. The implication of the particles’ ability to produce ROS is that the particles may have long-lasting effects in biological systems.

**Figure 3:**
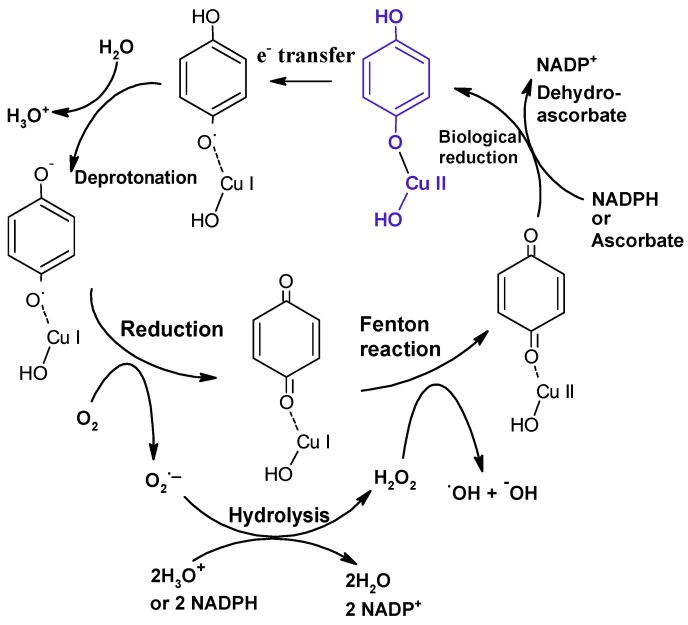
Proposed cycle for the generation of ROS by a pollutant-CuO particle system. The process begins when CuO is chemisorbed to the surface of the particle (Figure 2). For simplicity, in this example, hydroquinone is shown as an example of an organic pollutant. Beginning with the structure indicated in blue and working counter-clockwise, hydroquinone is adsorbed to the surface of the CuO-particle system and an electron is transferred from hydroquinone to Cu(II) to generate Cu(I) and semiquinone free radical. Following deprotonation of the other phenolic proton, an electron is transferred from the chemisorbed-radical compound to molecular O_2_ to produce superoxide and a non-radical product. Finally, the superoxide is converted to H_2_O_2_ in the presence of NADPH, ascorbate or thiols, all in high abundance on the lung surface. The resulting H_2_O_2_ can undergo Fenton-type reactions with the chemisorbed Cu(I) to produce·OH, and can regenerate Cu(II) on the particle to complete an ROS-generating redox cycle.

Although our colleagues have observed EPFRs in all ambient air PM_2.5_ samples examined to date [15,73], there are as yet no human exposure data. However, model pollutant-particle systems have been developed to study the biological effects of EPFRs, their ROS formation and their cytotoxicity [8]. These EPFR surrogates contain phenolic and/or chlorinated aromatic precursors (e.g., monochlorophenol or dichlorobenzene) adsorbed onto metal oxide domains bound to a fumed amorphous silica matrix. In *in vitro* studies, these EPFR surrogates were capable of generating ROS, including superoxide and the highly reactive hydroxyl radical, with a comparable yield to ambient air PM_2.5_ [64,68,74]. The measured cycle lengths for these particle systems indicate that they undergo numerous redox cycles [68]. Our hypothesis for EPFR-induced initiation of a redox cycle within a biological environment and their ability to generate ROS is illustrated in Figure 3. In support of this hypothesis, absence of any one of the components of the particle system, be it the transition metal or the organic, produced particles that generated little or no ROS, and the particles containing an EPFR were more toxic to cells *in vitro* than particles lacking an EPFR [68]. While a number of laboratories have reported that combustion-derived PM exposures elicit oxidative stress responses in both cultured cells and rodents [10,66,75,76,77], our studies utilizing model particle systems demonstrate that EPFRs produce greater levels of oxidative stress and overall toxicity compared to particles that do not contain an EPFR and are not themselves capable of producing ROS [66,68]. Studies using EPFR surrogates have also demonstrated their ability to induce both pulmonary and cardiovascular dysfunction in animals. In rodent models of asthma, EPFRs increased oxidative stress, dendritic cell activation and innate immune responses in the lungs of adult animals [78]. In neonates, EPFRs, but not size identical particles lacking an EPFR, induced airway inflammation and hyper-responsiveness, [79] as well as an increased severity of influenza disease [80]. Finally, EPFR-exposed neonatal mice exhibited an exacerbated allergic inflammation when challenged with allergen as adults [81]. With respect to cardiovascular toxicities, EPFR-exposed rats exhibited decreased cardiac function and increased oxidative stress at baseline, as well as after ischemia/reperfusion (I/R) injury [82]. EPFR inhalation also prevented the heart’s ability to compensate for deficits in left ventricular function induced by an IR injury [83]. These two findings may help to explain the association between PM exposures and mortality due to myocardial ischemic injury. Finally, in healthy rats, EPFR inhalation reduced left ventricular function associated with an increased oxidative stress, whereas exposures to particles lacking an EPFR exhibited no functional deficits or oxidant injury [84]. We have not yet specifically examined the impact of particle size on EPFR-mediated functional outcomes in animals, but given that the surrogate EPFR systems can be synthesized at varying sizes, such experiments are possible and likely warranted.

In conclusion, numerous published reports establish that EPFRs induce ROS production and biomolecule oxidation (Table 2), and several *in vivo* studies demonstrate that EPFRs exhibit greater toxicity than non-EPFR PM [79,80,84]. These outcomes suggest that adequate risk assessment of PM exposures will require a strategy for detecting EPFRs associated with PM and for estimating the contribution of EPFRs to disease risk. Therefore, a better understanding of EPFRs, their mechanisms of toxicity and careful risk assessment strategies for handling EPFRs appear in order.

## 6. Exposure Models of Toxicity—Challenges and Unresolved Questions

### 6.1. Particle Aggregation

It is now widely accepted that the toxicological study of nanoparticles either *in vitro* or *in vivo* is plagued by a number of methodological challenges. For example, do the nanoparticles actually exist as nanoparticles or as aggregated micron-sized particles? Even more important, to study their effects *in vitro* and *in vivo*, do we prevent them from aggregating? For example, if they do indeed aggregate, do the EPFRs interact to form new entities that themselves elicit cytotoxic properties, and should these new entities be studied? Nevertheless, most studies typically involve dispersal and suspension in solution with the aid of surfactants or other such agents to prevent their rapid aggregation. We typically utilize solutions of saline containing a small amount of Tween 80 and have found this surfactant to be relatively non-toxic to cells and not to contribute to ROS production. One may still wonder, though, whether we should be studying the monodispersed nanoparticles or the aggregates or even whether the use of a surfactant alters the mechanism of action of the particle system. Nanoparticles of many types are furthermore known to reduce the accuracy of cellular cytotoxicity measurements, as the particles typically either quench fluorescence or bind to reagents utilized in assays [85]. Moreover, nanoparticles are known to bind to cytokines, making the accurate assessment of cytokines through ELISA problematic [85]. Although we have found that many of these same challenges apply to the experiments utilizing EPFR-containing PM, as will be described below, the study of EPFR toxicity is associated with numerous other unresolved challenges over and beyond that of other nanoparticle exposures.

### 6.2. Particle Storage

A critical factor that cannot be overlooked is the storage of particles. Our prior data has shown that certain radical species associated with PM survive for months after extraction from the environment [54]; however, some radicals are less stable (*i.e.*, hours/minutes). Furthermore, suspension in solution for the sake of creating monodispersed nanoparticles can result in quenching and thus, their storage as dry particles under vacuum is important for maintaining their shelf life [8]. Thus, PM experiments conducted with less than optimal storage conditions likely culminates in EPFR quenching prior to experimentation and may not reflect the exposure effects associated with an EPFR, per se. Since filter systems designed to trap airborne PM typically generate only small quantities of particles over short periods of time, extending the collection time—*i.e.*, days to a few weeks—likely results in the quenching of radical collected in the beginning of the collection period. Consequently, it is hard to conceive of collecting sufficient EPFR-containing PM in a pristine condition for performing animal studies. In our own experience, studies such as these were restricted to *in vitro* designs only. Thus, it is likely that animal exposure studies conducted to date that assess the toxicity of PM collected from biological sources poorly assess the contribution of the EPFR. Thus, risks associated with PM exposures may be underestimated.

### 6.3. Use of Controls

Given that PM exposures themselves, independent of the EPFR, can elicit tissue responses such as inflammation, design and utilization of appropriate controls is important, but is also frustratingly difficult to achieve. Our research team has addressed a number of our experimentation issues by developing model EPFR [8,66]. Our laboratory-generated pollutant-particle systems have certainly overcome the issue of obtaining sufficient pristine EPFR-containing PM samples for exposing animals. However, design of the most appropriate surrogate PM for use as a control has proven problematic. For example, although we have sometimes utilized an EPFR surrogate system containing only a transition metal such as Cu(II) as a control, Cu(II) is itself a known oxidant in biological systems [86]. Considering that it may be unlikely to encounter a particle in the environment that contains only copper, and given that the Cu(II)-particle control will likely present with toxicity but with a differing mechanism of action, it seems unlikely that such a surrogate serves as an ideal control. One might also utilize, for example, amorphous silica as an appropriate control having a similar particle size but lacking an EPFR. Although a reasonable solution, silica does not contain an organic component that together with the EPFR, may be necessary for promoting biologic effects. To date, we have attempted to utilize a number of similar surrogate systems as controls [66,68], but despite our efforts, have yet to identify an ideal control for biological studies.

### 6.4. Mixtures

PM_2.5_ is a complex mixture of particles, along with sulfate, nitrates, ammonium, elemental carbon, metals, and organic carbon [87], as well as biological components such as lipopolysaccharide [88]. Epidemiological studies suggest that these components may exacerbate cardiopulmonary disease onset or progression. For example, as reviewed by Guarnieri and Balmes [1], NOx may potentially act as an adjuvant, such that combinations of PM and NOx interact to promote asthma symptomology or onset. Moreover, ozone has been shown associated with allergic sensitization [89]; thus, ozone may further exacerbate PM-induced asthma presentation. Therefore, animal experimentation should address not only the impact of PM exposures and the role of EPFR, but also the synergistic/antagonistic effects of EPFR-containing PM mixtures with other components of air pollution. It is possible that the numerous components synergize to elicit an increased risk of cardiopulmonary disease that may be underestimated from individual exposure studies.

In summary, exposure to elevated levels of combustion derived PM has been associated with adverse cardiopulmonary events. EPFRs exist on airborne PM_2.5_ at concentrations that are high compared to most organic pollutants (~1–10 μM/g). Traditional methods of analyzing PM (*i.e.*, solvent or alkaline extraction) result in their conversion to molecular species that may or may not contain an EPFR [90], suggesting that EPFRs are being misidentified as molecular pollutants or worse, not being detected at all. Thus, we believe that EPFRs represent a new paradigm for the human health impacts of environmental PM and that risk assessment for EPFRs is timely and critical.

## 7. EPFRs and the Regulatory Framework

Since EPFRs are a new class of pollutant, policy makers in the U.S. have not formulated specific regulations to address the potential public health risks. However, existing policy governing the incineration of hazardous materials and the Clean Air Act’s standards for fine particulate matter predict how EPFR regulation might be addressed.

### 7.1. Incineration of Hazardous Materials

Emissions from hazardous waste incinerators are regulated under the Clean Air Act (CAA). The policy is implemented by the states through permitting of hazardous waste incineration operations. Under the existing policy, states may adopt more stringent permit requirements than those required by the CAA. The CAA is designed to protect human health and the environment from the most harmful effects of air pollution by requiring significant reductions in the emissions of the most dangerous air pollutants. These pollutants are known or suspected to cause serious health problems such as cancer or birth defects, and are referred to as hazardous air pollutants (HAPs).

Under the original CAA, the Environmental Protection Agency established National Emission Standards for Hazardous Air Pollutants (NESHAPs) for seven HAPs. The 1990 amendments to the CAA required that the standards be based not on a specified level, but on the maximum achievable control technology (MACT) for a category of emission sources within an industry group, such as hazardous waste incinerators. The policy applies an environmental policy tool or instrument known as “design standards” and requires that the regulated entity apply the technology used by the most successful pollution-reduction firm within an industrial category.

The specific regulations for hazardous waste incinerator operators are found in the Code of Federal Regulations, Part 264, Subpart O. (USEPA website). Permitted incineration facilities are required to conduct risk assessments to ensure that the incineration process does not emit materials that may pose a public health threat. EPA sets a relatively stringent standard for hazardous waste incinerators at 10 times more protective than the allowable limits of the same substances involved in other permitted processes. The required risk assessment must include direct and indirect potential pathways of human exposure. Direct pathways include inhalation and ingestion, while indirect routes include deposition on soil and in waterways, leading to possible introduction of the material into the food chain. Under current regulations, the estimated emissions must account for any metals, dioxin, and other products of incomplete combustion (PICs) that may be present. There is as yet no requirement for EPFR monitoring.

### 7.2. Clean Air Act (CAA) Regulation of Particulate Matter

The current version of the CAA also provides guidelines and protocols for limits on PM_10_ and PM_2.5_, based on their physical properties as pulmonary irritants. The EPA recognizes the ability of these coarse (PM_10_) and fine (PM_2.5_) particles to decrease lung function, aggravate existing asthma, and induce chronic bronchitis, irregular heartbeats, and nonfatal heart attacks. All of these health effects are based solely on the mechanical properties of particulates small enough to enter and irritate airways and alveolar sacs. However, ultrafine particles (PM_0.1_) have recently been shown to cause much more severe problems [91,92]. As a percentage of mass, these particles only make up a small portion of the total particulate assemblage, but as a percentage of the total number of particles, the ultrafines comprise up to 90% of the assemblage [91]. However, it is already widely appreciated that the ultrafine particles are capable of greater penetration into lung tissues. Nevertheless, many unknowns concerning the intersection of ultrafines and EPFRs remain. For example, it is unknown whether EPFR concentrations are greater in ultrafines compared to PM_2.5_ or PM_10_. Moreover, given the findings of our studies [66,68], EPFR-containing ultrafine particles present with greater toxicity than those absent an EPFR. Thus, the contribution of EPFR cannot be overlooked in either strategies for risk assessment or for the development of additional policies for regulating PM exposures.

What is the outlook for more specific or stringent policy guidelines in the future? Public policy theory offers insights. Kingdon’s conceptual model of policy “agenda setting” [93] describes the process by which issues are recognized as important enough to warrant a policy response. The first component of the process is the “problem stream”, wherein scientific certainty concerning risks associated with a substance is high and public sentiment supports a policy response to address the risk. Often, a “triggering event” is necessary to convince observers that the hazard is significant and that the regulatory status quo is not adequate. Next, policy approaches recognized as appropriate and effective means for addressing the problem, ranging from direct command-and-control regulations to incentive-based programs, are designed to promote best practices [94]. Finally, solutions to the problem are typically defined largely by the responsible government agency and regulated entities that would bear increased costs associated with new or more stringent regulation. Affected economic interests are more likely to accept new regulation if they believe the regulations would be implemented equitably without bias throughout the industry, and would not introduce unreasonable costs or onerous reporting requirements [93,95]. As a result of the inherent difficulties of achieving movement within these policy realms, many environmental concerns do not advance to the public policy agenda.

Applying this descriptive model to the case of EPFRs and PM_0.1_, federal funding opportunities and recent research have increased awareness of the issues and likely risks, at least among the research community and government agencies. However, there is significant uncertainty concerning the extent to which the general public is subject to exposure risks. The dearth of research to date concerning the contribution of EPFRs to PM-associated health effects, and the lack of an ability to measure EPFRs in real-time hinder progress toward scientific certainty. As a result, the “problem stream” for EPFRs is not well-defined, and advancement through the public policy agenda is not yet supported.

## 8. Conclusions

The discussion points introduced here provide a context for the consideration of additional research and public policy actions to address EPFRs, an important emerging contaminant. The lack of scientific certainty concerning the extent to which EPFR’s may pose risks to public health and our lack of an ability to measure them in real-time are major impediments to accurate risk assessments and new guidelines to address the threat. As also outlined here, another critical need is the development of risk assessment strategies for EPFR-containing PM, and this can only happen once substantial *in vitro* and *in vivo* experimentation has been completed.

A feasible next step concerning EPFR’s would be to work within the existing policy framework to promote additional monitoring, environmental sampling for EPFRs around permitted hazardous waste incinerators, and studies aimed at mapping human exposures around these sites. This could provide a key source of data to support more refined estimates of the location and extent of human exposure risks associated with EPFR’s. This information along with a better understanding the science and development of technologies that could minimize the risk of EPFR formation in the incineration process could be implemented within the MACT program to reduce public exposure risks associated with this emerging contaminant.

## Figures and Tables

**Table 1:** Literature support for the formation of EPFRs formed through the combustion of organic materials.

Source Material	EPR Signal (*g* Value)	(Free Radical) (Spins/g)	Reference
Wood (fatwood, pine wood) Coal (bituminous and anthracite)	2.0029–2.0035	2.3 × 10^17^–1.2 × 10^18^	[12]
Biochar (pine needles, wheat straw and maize straw	2.0028–2.0037	1.96–30.2 × 10^18^	[11]
DEP, GEP, woodsmoke, cigarette tar, and airborne PM ^1^	2.0025–2.0040	10^15^–10^17^	[13]
TSP (Athens), Urban street dusts, DEP, GEP	2.0036 (single, broad signal)		[16]
DEP	~2.0		[17]
Polymer: PS, PVC, PE, PP, PET	2.0028–2.004	2 × 10^12^–8 × 10^13^	[14]

^1^: Collected as total suspended particulate at <0.3 μm in Athens, Greece. Samples were found to contain trace metals, including iron, copper, zinc, vanadium, nickel, chromium and magnesium. Abbreviations: DEP = diesel exhaust particles; GEP = gasoline exhaust particles; PS = poly(styrene); PVC = poly(vinylchloride); PE = poly(ethylene); PP = poly(propylene); PET = poly (ethylene terephthalate), TSP = total suspended particulates.

**Table 2:** Evidence for the ability of EPFRs to generate reactive oxygen species.

Source	Finding	References
TSP (Athens); Urban street dusts; DEP; GEP	PM generates hydroxyl radical in aqueous suspension. Hydroxyl radical formation was linked with redox-active metal content.	[72]
Biochar	Biochar contains persistent free radicals evident by EPR. Biochar can activate H_2_O_2_ to produce hydroxyl radical.	[11]
DEP; Coal fly ash	Suspensions of DEP and coal fly ash produce hydroxyl radical. Metal ions and superoxide implicated in its production. Neither kaolinite nor silica produce ·OH.	[69]
Ambient air PM (California); DEP	In the presence of ascorbate, ambient air PM and DEP both generate ·OH. ·OH production is correlated with Cu content	[70]

Abbreviations: DEP = diesel exhaust particles, GEP = gasoline exhaust particles, TSP = total suspended particulates.

## Abbreviations

The following abbreviations are used in the manuscript:

CAA	Clean Air Act
EPFR	Environmentally persistent free radical
EPR	Electron paramagnetic resonance
DEP	Diesel exhaust particles
GEP	Gasoline exhaust particles
PE	Poly(ethylene)
PET	Poly (ethylene terephthalate)
PM	Particulate matter
PM_10_	Particulate matter (diameter < 10 μm)
PM_2.5_	Particulate matter (diameter < 2.5 μm)
PP	Poly(propylene)
PS	Poly(styrene)
PVC	Poly(vinylchloride)
ROS	Reactive oxygen species
TSP	Total suspended particulates
